# Ugandan stakeholder hopes and concerns about gene drive mosquitoes for malaria control: new directions for gene drive risk governance

**DOI:** 10.1186/s12936-021-03682-6

**Published:** 2021-03-16

**Authors:** Sarah Hartley, Robert D. J. Smith, Adam Kokotovich, Chris Opesen, Tibebu Habtewold, Katie Ledingham, Ben Raymond, Charles B. Rwabukwali

**Affiliations:** 1 University of Exeter, Northcote House, Queen’s Drive, Exeter, EX4 4QJ UK; 2 Science, Technology & Innovation Studies, School of Social and Political Sciences, University of Edinburgh, Chisholm House, High School Yard, Edinburgh, EH1 1LZ UK; 3 Department of Forestry & Environmental Resources, Genetic Engineering and Society Center, North Carolina State University, Campus Box 7565, Raleigh, NC 27695-7565 USA; 4 Makerere University, PO Box 7062, Kampala, Uganda; 5 Department of Life Sciences, Imperial College London, Exhibition Road, London, SW7 2AZ UK; 6 University of Exeter, Penryn Campus, Treliever Road, Penryn, TR10 9FE UK

**Keywords:** Malaria control, Gene drive mosquitoes, Uganda, Stakeholders, Risk governance, Risk assessment, Target Malaria

## Abstract

**Background:**

The African Union’s High-Level Panel on Emerging Technologies identified gene drive mosquitoes as a priority technology for malaria elimination. The first field trials are expected in 5–10 years in Uganda, Mali or Burkina Faso. In preparation, regional and international actors are developing risk governance guidelines which will delineate the framework for identifying and evaluating risks. Scientists and bioethicists have called for African stakeholder involvement in these developments, arguing the knowledge and perspectives of those people living in malaria-afflicted countries is currently missing. However, few African stakeholders have been involved to date, leaving a knowledge gap about the local social-cultural as well as ecological context in which gene drive mosquitoes will be tested and deployed. This study investigates and analyses Ugandan stakeholders’ hopes and concerns about gene drive mosquitoes for malaria control and explores the new directions needed for risk governance.

**Methods:**

This qualitative study draws on 19 in-depth semi-structured interviews with Ugandan stakeholders in 2019. It explores their hopes for the technology and the risks they believed pertinent. Coding began at a workshop and continued through thematic analysis.

**Results:**

Participants’ hopes and concerns for gene drive mosquitoes to address malaria fell into three themes: (1) ability of gene drive mosquitoes to prevent malaria infection; (2) impacts of gene drive testing and deployment; and, (3) governance. Stakeholder hopes fell almost exclusively into the first theme while concerns were spread across all three. The study demonstrates that local stakeholders are able and willing to contribute relevant and important knowledge to the development of risk frameworks.

**Conclusions:**

International processes can provide high-level guidelines, but risk decision-making must be grounded in the local context if it is to be robust, meaningful and legitimate. Decisions about whether or not to release gene drive mosquitoes as part of a malaria control programme will need to consider the assessment of both the risks and the benefits of gene drive mosquitoes within a particular social, political, ecological, and technological context. Just as with risks, benefits—and importantly, the conditions that are necessary to realize them—must be identified and debated in Uganda and its neighbouring countries.

## Background

Gene drive mosquitoes are an important emerging technology for vector control. The yearly downturn trend in malaria cases and deaths has plateaued since 2015 [1]. This stagnation in control is caused by insufficient coverage with insecticide-treated nets and indoor residual spraying as well as selection for mosquito populations that mediate outdoor malaria transmission and the prominence emergence of vectors with multiple insecticide resistance [1]. Gene drive mosquitoes may be an additional tool to complement nets and spraying by blocking the transmission of *Plasmodium* to humans. In 2020, the African Union’s High-Level Panel on Emerging Technologies singled out gene drive mosquitoes as one of three priority technologies to contribute to malaria elimination [2]. The complexity of gene drive technology, its reliance on the rapid spread of genetically engineered elements and its potential to persist in environments and transform ecologies means that this topic is receiving increased attention in the social and natural sciences [3–6]. The first field trials of gene drive mosquitoes are expected in 5–10 years in Uganda, Mali or Burkina Faso [7, 8]. These mosquitoes are likely to be the first gene drive organisms field-tested anywhere in the world and, therefore, place a spotlight on scientific developments and risk governance in sub-Saharan Africa.

A gene drive is a mechanism that increases the frequency of a desired gene in a mosquito population by increasing the rate at which it is spread through reproduction [9]. Combining gene drive with genome editing techniques, scientists are able to genetically modify the *Anopheles* mosquito genome and push modifications through the natural mosquito population to either suppress the population or replace it with genetically engineered mosquitoes [10]. This contrasts with many genetically engineered products in which the spread of genetically engineered constructs in a target species is generally unwelcome.

Existing risk governance frameworks for genetically engineered organisms seek to avoid the environmental spread of introduced genes. This means that in order to prepare for field trials of gene drive mosquitoes, existing risk and regulation frameworks need to be evaluated and it is likely that risk governance guidelines must be developed or modified. Traditional field trials, which rely on controlled releases in specific geographical sites, will be difficult as gene drive constructs are designed to spread rapidly and may not respect the boundaries of a trial site [11]. The gene drive developer community, made up of researchers, funders, supporters, regulators and policy-makers, has begun to delineate a governance framework, identifying potential risks arising from the development, testing and possible deployment of gene drive mosquitoes and determining how to manage them. These efforts involve expert workshops, meetings, academic papers and draft guidance [7, 12–14].

Scientists and bioethicists have raised concerns about the small number of African scientists, stakeholders and publics involved with gene drive mosquito development and governance [4, 15]. Echoing these concerns, African scientists have called for the involvement of a broad range of African scientists and stakeholders in conversations about gene drive mosquitoes for malaria control [16, 17]. African policy-makers have joined these calls, arguing that the knowledge and perspectives of those people living in malaria-afflicted countries is missing in such conversations [17, 18]. Lastly, bioethicists argue that ethical debate about whether to release gene drive mosquitoes in field trials should include African stakeholders living in malaria endemic areas [15].

Despite these calls for the involvement of African scientists, stakeholders and communities in gene drive development and governance, very few efforts to develop risk governance frameworks have actively sought to engage African publics. Many workshops to explore risks of releasing gene drive mosquitoes in Africa have engaged European or American stakeholders [7]. Even where international workshops were designed to explore potential risks related gene drive mosquitoes for malaria control in sub-Saharan Africa, African participants were largely absent in comparison to European and American participants (Europe/USA = 34, Africa = 8, Asia/South America = 3, WHO = 2) [12]. One of the few exceptions was a series of four regional consultations organized by the New Partnership for Africa’s Development and held in Ghana, Kenya, Botswana, and Gabon during 2016–2018 [6]. Here, African stakeholders identified similar protection goals and harm pathways to American stakeholders at an earlier workshop in 2016 but also raised new concerns about mosquito behavior and fresh water protection goals [6].

The very limited African input into decisions about how to govern gene drive mosquitoes for malaria control has led to explicit calls for empowering and engaging African stakeholders who understand the local health, social-cultural as well as ecological context [12]. Neves and Druml [15] emphasize the importance of ‘*reflecting on the risks and benefits for people living in areas endemic for malaria*’. Engaging with African publics is likely to bring substantive insights to risk governance which may not be identified by other actors [19]. It may also improve the value of risk governance by making it meaningful and informative [7]. Situating engagement in the local context is essential for risk governance, not only to capture the specific technology application being used and the receiving environment, but to ensure that local political context and local values inform the many value judgments inherent to the risk governance process [20, 21]. Taitingfong has argued:

“*Because concepts such as risk and benefit are contingent upon the communities or persons defining them, it is important that decisions surrounding gene drive do not rely solely on extant frameworks that will inevitably omit culturally specific considerations, including those of indigenous groups*” [22].

Gene drive technology’s unique risks (namely, to spread throughout a population, persist in the environment, and cause irreversible effects) and ethical concerns about developers from high-income countries shaping risk governance frameworks in low- and middle-income countries further compel the need to ensure that contextually-specific values, knowledge, hopes and concerns feed into the development of risk frameworks. This lack of knowledge about what questions need asking and how they are answered in relation to the local context for governance of gene drive mosquitoes presents a critical gap. This study brings the local context to bear on this promissory technology for malaria control by investigating and analyzing the hopes and concerns that matter for Ugandan stakeholders and mapping out the new directions needed for gene drive risk governance in light of the results.

## Methods

### The study setting

Uganda has one of the highest incidences of malaria in the world and malaria remains the leading cause of death [23, 24]. Currently, malaria is controlled in Uganda through indoor residual spraying, drug treatments and the provision of free insecticide-treated bed nets—the Ministry of Health recently launched a new universal campaign to distribute over 27 million nets across Uganda [25]. However, the ongoing pervasiveness of the disease points to the need to consider innovative solutions as part of a broader toolkit for malaria control. Target Malaria, a multinational research consortium funded largely by the Bill and Melinda Gates Foundation (BMGF) (US$75 million) and the Open Philanthropy Project (US$17.5 million), is expected to conduct the world’s first gene drive trial in Uganda, Mali and/or Burkina Faso [8]. Target Malaria’s Ugandan team is based at the Uganda Virus Research Institute (UVRI), a medical research institute located in Entebbe and known for its entomological expertise. Target Malaria has allocated funds to build scientific capacity and a new insectary at UVRI. Mosquitoes are collected from villages on islands in Lake Victoria and in Mukono and Kayunga districts to provide baseline research data and Target Malaria now has regulatory approval for importation and contained use of gene drive mosquitoes in Uganda [10].

Uganda is a signatory to the Convention on Biological Diversity and the Cartagena Protocol on Biosafety. In 2018, the Parliament passed the Genetic Engineering Regulatory Bill which contains a strict clause on liability and redress to hold gene drive developers liable if harm results from field trials. However, in 2019, the President announced his refusal to assent to the Bill for the second time, explicitly mentioning genetically modified mosquitoes and citing several concerns about safeguarding citizens and the ecology stating that ‘*commercial interests, however, need to be balanced against the need to protect the ordinary Ugandan Citizen from real and potential harm, health and wellbeing rather than profit, must be our primary concern*’ [26]. When Target Malaria is ready for field trials, it will seek approval from the National Environmental Management Authority, Uganda Virus Research Institute Biosafety Committee, National Biosafety Committee at the Ugandan National Council for Science and Technology, and National Bioethics Committee at the Ministry of Science, Technology and Innovation. Regionally, the BMGF and the Open Philanthropy Project, Target Malaria’s funders, provide considerable funds to the African Union Development Agency (AUDA) to build regulatory research capacity in Africa and prepare for the testing and deployment of gene drive mosquitoes for malaria control [27, 28]. AUDA is committed to developing a harmonized approach to regulation and provides significant support to national regulators in collaboration with Target Malaria. Its goal is to ‘*create enabling regulatory environments that allow science, technology and innovation to thrive*’ explicitly for malaria control applications [29].

### Data collection

Data was collected between August and December 2019 using semi-structured interviews with 19 key Ugandan actors including one social scientist, four entomologists/biotech experts, two medical doctors, one veterinary doctor, two representatives of the community and/or members of the vulnerable and marginalized groups living near to UVRI, five biotechnology experts, one legal expert, one environmentalist and two biosafety experts. Participants were selected for their knowledge of, or experience with gene drive mosquitoes for malaria control and/or Uganda’s governance framework for this technology. Individuals from a breadth of backgrounds were included to capture a comprehensive range of perspectives and interests. For example, two participants claimed to be opponents of gene drive, eight were proponents and seven were self-identified as neutral.

Following established social science methods, interviews were semi-structured and structured around the following questions: What hopes do you have for this technology?; What concerns do you have about this technology? Interviews explored participants’ hopes for the technology, the concerns they believed pertinent, and the ways in which these concerns might be addressed [30]. Due to the contentious nature of biotechnology in Uganda, some participants felt reluctant to engage and considerable effort was placed on building trust prior to and in the course of the interview. Some targeted participants in regulatory institutions were not cleared to participate in the interviews and others kept postponing appointments until the study ended. While most interviews were recorded and transcribed, a few participants refused permission for their interview to be recorded. Here, detailed notes were taken instead. The research received ethical approval by Makerere University Social Sciences Research and Ethics Committee (MAKSS REC 05.19.300) and National Biosafety Committee of the Uganda National Council for Science and Technology (SS5059). All participants provided consent to the use of anonymous quotes in research outputs.

### Data analysis

Initial, explorative, analysis was conducted at an interdisciplinary coding workshop in December 2019 in the UK and comprising all authors. Following the workshop, four authors (SH, RS, AK, CO) coded the transcripts thematically [31]. All portions of text were extracted using the phrase ‘*hopes and concerns associated with gene drive development and deployment*’ as a heuristic to guide the data reduction process. Similar terms such as ‘*risk and benefit*’ were deliberately not chosen to guide this process to avoid imposing external categories and meanings onto participants’ discourse. Data were organized into coherent categories, ultimately resulting in the three overarching themes. To ensure consistency in coding, two coders (RS and SH) completed the process individually and divergences were discussed and revised. The outcomes of the coding process were then presented to the remaining pair of the analysis group (AK and CO) to ensure coherence and comprehensiveness.

## Results

### Stakeholders’ hopes and concerns about gene drive mosquitoes

Participants’ hopes and concerns for gene drive mosquitoes to address malaria fell into three broad themes: (1) ability of gene drive mosquitoes to address malaria, highlighting the importance of efficacy; (2) impacts of gene drive testing and deployment, highlighting the consequences associated with gene drive development and use; and, (3) governance, which is concerned with the processes of governing and developing the technology democratically. Stakeholder hopes fell almost exclusively into the first theme while concerns were spread across all three. Table 1 summarizes these findings. Each of these three themes and their sub-themes are presented below and illustrated with participant quotes.

**Table 1:** Ugandan stakeholders’ hopes and concerns for gene drive mosquitoes for malaria control

Hope sub-themes	Themes	Concern sub-themes
***Effective at reducing malaria*** ***Another possible tool in the fight against malaria***	Ability of gene drive mosquitoes to address malaria	***Lack of public and political support hinders development and deployment*** ***Technological intervention is ineffective***
***Ecological impacts*** Less environmental impact than chemical pesticides ***Socio-economic impacts*** Increase in productivity and cost-savings	Impacts of gene drive testing and deployment	***Ecological impacts*** Decrease in non-target organisms and/or species diversity as the elimination of the mosquito species reverberates through ecosystem Gene drive construct spreads to, and negatively impacts, non-target organisms through vertical or horizontal gene transfer ***Human health impacts*** Emergence of new diseases and/or increase in the prevalence of existing diseases Negative impacts on human health ***Socio-economic impacts*** Negative impacts on the economy and society Costs and benefits unfairly distributed
	Governance of gene drive mosquitoes	***Novelty and irreversibility*** High risk of irreversibility, uncertainty and lack of experiences create governance challenge Excitement and hype crowd out alternative approaches to malaria reduction ***Inter-state relations*** Political conflict between neighbouring African nations ***Institutional uncertainty*** Unclear regulatory pathway and lack of regulatory capacity Lack of transparency and independence Lack of clarity on liability and responsibility

#### Theme 1: Ability of gene drive mosquitoes to address malaria

The overwhelming hope stakeholders identified was to reduce or eliminate malaria. Within this theme, two sub-themes emerged. The most frequent is that gene drive mosquitoes are *effective at reducing malaria* (U1–U11, U14–U18):

“*We are all optimistic that this will succeed and we shall have a big breakthrough in reducing malaria transmission in all the malaria endemic countries*.” (U2)

Participants highlighted the significant burden of malaria in Uganda noting it is one of the biggest killers of children, women and others in Uganda and across Africa:

“*Malaria’s been our biggest killer. Our children, our women, our people*.” (U6)

Some participants imagined that gene drive mosquitoes could eliminate malaria and result in a type of utopia:

“*If there’s no malaria being reported in a certain municipality where the mosquitoes are being released, for a period of six months, then the people will say, ‘Hey, what is so special about that municipality?’ Then everyone will know, ‘Oh, they released genetically*-*modified mosquitoes.’ People will start talking that, ‘Oh, actually, now I’m having good nights. I’m not being bitten by mosquitoes anymore. We used to buy chemical pesticides, we used to buy coils to smoke the house, we used to go to the hospital every now and then* – *now we are not going.*’ *Statistics in hospitals will show that there’s no more malaria, and what else do you want in life? That’s the perfect environment you would like to stay*.” (U9)

Other participants noted the possible increase in vector-borne diseases as a result of climate change and hoped that gene drive mosquitoes could also be an adaptation tool:

“*Malaria is a very big issue in Uganda and having something which can actually prevent the multiplication of mosquitoes may have a big impact in trying to reduce the cases of malaria, and also there is the issue of climate change* - *we expect increased temperatures and maybe new types of malaria and the mosquitoes that migrate and perhaps evolve*.” (U7)

The second sub-theme of hopes is that gene drive mosquitoes are *another possible tool in the fight against malaria* (U1-U6, U8-U9, U18-19). Some participants argued that gene drive mosquitoes might be better than some of the alternatives because they are expected to be low cost (U2), while others believed gene drive mosquitoes are more targeted on the vector than alternatives and therefore better at reducing malaria:

“*Instead of addressing the problem from the symptomatic side it addresses it at the source*.” (U5)

Some participants believed that gene drive mosquitoes could be better than alternatives in reaching hard-to-reach, poorer communities because they could bypass the need for human involvement:

“*There are other tools that have been brought up lately, mosquito nets, malaria drugs, but they have turned out to be not very effective because of the conception of the people. That’s why* [gene drive] *has been introduced, with the thinking that when they let the mosquito out, a particular type of mosquito with some effect, it will do its work without even the knowledge of the community people*.” (U8)

For others, the ability for gene drive mosquitoes to reach hard-to-reach, poorer communities meant that those with little resources would benefit from malaria reduction or elimination.

“*For Africa, which is very rural, where 80% of the population who live in the rural areas cannot access these chemicals for spraying to deal with a vector like malaria among mosquitoes, they are constantly going down with malaria. They don’t have medicine. As the [mosquito] population declines, the people are made free from the disease and they are more productive. They are also productive in the sense that they don’t fall sick, spend their little resources on drugs, because there’s something controlling the population of the enemy. So I think this technology can be used in the situation where you have the poorest of the poor being the key beneficiaries*.” (U9)

The ability of gene drive mosquitoes to reduce or eliminate malaria was not only the overriding hope but also a source of concern about the possible failure of gene drive to reduce or eliminate malaria. This concern has two sub-themes. The first and dominant sub-theme was that gene drive does not reduce malaria because a *lack of public and political support hinders its development and deployment* (U1-3, U5, U8-9, U15-16, U18). The most significant barrier was seen to be public suspicion or fear of new technologies and of GMOs specifically:

“*One of the major challenges* [gene drive] *faces is the acceptability from the public, the public opinion goes way against it. There is a lack of understanding by the community on exactly what biotechnology is and what it does. Most of them are benefiting from the products but if you introduce the same products and say this is what you are consuming, very many of them would reject*.” (U15)

Factors such as strict liability clauses that allocate liability to the developers of technology were seen as strong disincentives for development of a transformative technology. Participants were concerned that public or political suspicion—and a failure to allay it—would foreclose a transformative technological trajectory to address malaria:

“*I’m convinced that gene drive is a sure way of eliminating malaria, it’s a sure way … Now if it is exposed to the laws that have restricted the other GMOs it might cut us from having the sure way of eliminating malaria. It has been very hard to convince these bodies to implement it here … Even if we know the results will be good, it can be stopped*.” (U8)

The second subtheme to emerge is that gene drive mosquitoes do not reduce malaria because the *technological intervention is ineffective* (U2, U4, U5, U6, U7, U13, U15). For instance, resistance to the trait or the drive may develop, removing the mosquitoes may reduce the ecological burden placed on other malarial vectors, or the environmental fitness of the modified mosquitoes may be too poor to be sexually competitive, meaning that the trait would not spread:

“*Malarial mosquitoes are so different depending on where it is. I hope those are Ugandan type mosquitoes that were from here and you’re just deploying them back because if you bring another* – *it’s like when you bring Friesians* [cattle]*, which do well in Europe, cold nice temperature, put them here… they’ll just go down, they find disease*.” (U6)

#### Theme 2: Impacts from gene drive testing and deployment

Stakeholders identified a variety of hopes and concerns related to the potential impacts of testing and deployment of gene drive technology to address malaria which fell into three sub-themes. The most frequent of these was potential *ecological impacts* of a gene drive mosquito (U1-8, U10-16, U18). Here, concerns largely fell into two sub-categories: 1) a decrease in non-target organisms and/or species diversity as a result of the reduction in mosquitoes, and 2) potential negative ecological impacts as a result of the gene drive construct spreading through vertical or horizontal gene transfer. Participants often articulated concerns about the impacts on non-target organisms and species diversity in the specific Ugandan setting, pointing to the importance of local context when identifying priorities for risk assessment:

“*We have animals that feed on mosquitoes, what will those animals feed on? In Uganda, bats and chameleons, so they might also disappear*.” (U8)

Although acknowledged as relatively unlikely, participants also identified as a concern the potential for the gene drive construct to move across species:

“*These genes are not just passive… Hopefully it doesn’t jump from one phylum to another one, using all kinds of variation. They’re not supposed to, but genes can find their way through all kinds of ways and you find them going there. So untargeted organisms receiving these novel sets of genes and what happens to them will continuously be [the] question*.” (U6)

While this sub-theme of ecological impacts was dominated by concerns, some participants were hopeful about the ecological impacts too, pointing out that gene drive mosquitoes were preferable because they will have less environmental impact than chemical pesticides:

“*If we can have a biological method of control in the form of gene drives, you will find out that there is less need for harmful pesticide, which has a myriad of consequences. Environmentally, you have reduction of beneficial organisms like bees getting colony collapse disorder when you use less pesticide. As well, you use less energy in the creation of these pesticides, and you can reduce your carbon footprint because you have less need for actually making those types of pesticides.*” (U19)

Many participants also articulated concerns involving *human health impacts* (U2, U4, U6, U8, U11, U13-17). These concerns predominantly fell into two sub-categories: 1) an increase in new diseases and/or the prevalence of existing diseases, and 2) a general concern about potential negative impacts from gene drive mosquitoes on human health. For instance, one participant expressed concern about how a replacement gene drive could potentially lead to other diseases:

“*Maybe they become efficient vectors of another disease that was not a problem, and then yes you dealt with the malaria, it’s no more, but you have made them now transmit something else that you get*.” (U4)

Participants also discussed how a potential increase in malaria prevalence could result from the interaction of different mosquito species in the Ugandan context. One participant expressed concern about how a gene drive-caused decrease in one mosquito species could lead to the competitive release of another mosquito species that is also a malaria vector, thereby not resulting in a reduction in malaria:

“*In Uganda the most common vector which is responsible for over 70% of malaria transmission is the* Anopheles gambiae*. So that is why we were saying that the other vectors do not thrive as well as the* Anopheles gambiae*. So the possibility that the* Anopheles gambiae *causes a burden, an ecological burden on the other vectors, so that if you remove it then the others will thrive. So that might not favor our ultimate aim of reducing malaria transmission.*” (U2)

Participants also stated general concerns about gene drive impacting human health. One participant described a concern about whether a gene drive, designed to impact the genome of an entire species, could end up impacting the human genome.

“*So, I think the fear is the unknown probably at this stage… If you can affect the mosquito genome, can it affect the human genome*?” (U11)

Another participant shared a general concern about the impact of a gene drive on human health because of the interconnectedness of humans and their environment:

“*Human beings by their nature use what is in the environment for survival, food, everything, so whatever comes in that ecological set*-*up ends up in the human body, one way or the other. So that is another angle that some people raise issues about, and perhaps that’s what scares people most*.” (U14)

The third sub-theme involves hopes and concerns about the potential *socio*-*economic impacts*, particularly potential changes to established social, cultural and economic practices and organizations resulting from the development or deployment of gene drive mosquitoes. Stakeholders raised a wide range of concerns beyond human health and environmental risk (U1-2, U5, U7-8, U10, U13-14, U16):

“*This issue of gene drive is not just about science, it’s about human rights, it’s about social cultural aspects*.” (U10)

The specific issues raised were diverse including potential impacts on the communities living around test sites, with one participant questioning whether Ugandans were being used as guinea pigs (U2). Another participant suggested that inadequate consideration and management of community relations could result in an exodus from these villages. Others were concerned with the impact of gene drive on entities that were central to Ugandan identity. For instance, when asked about the social impacts of gene drive use, one participant raised concerns about the effect on Lake Victoria, which has significant cultural as well as economic importance to Ugandans (U10). Several participants were concerned with the economic impacts of using gene drive to successfully eradicate malaria, emphasizing that while it may be a worthy goal there would be large-scale social and economic change, and within that change there would be losers as well as winners:

“*There are many people surviving on malaria, selling drugs, malaria pesticides to control mosquitoes and all those would be jobless and that would disorganize the world*.” (U1)

A related and shared concern was with the distribution of benefits. Instead, they suggested that the primary beneficiaries might be the scientists developing the technologies, who might publish and gain academic credentials, and raised concerns about whether local communities would benefit (U16). Drawing on a history of past development projects, one participant emphasized,

“*We have seen a lot of scientific innovations that have not benefitted the people, young or old and all of those kind of things. We have seen them but they have not benefitted the people*.” (U13)

However, some participants highlighted possible socio-economic benefits (U3, U5-7, U14, U18). Participants pointed to possible increases in productivity and cost-savings for the Ugandan, regional and local governments and individual households.

“*Less malaria, less death will affect the economy and the health of the people, because we know malaria is a really big budget in our country*” (U18)

“*You would think the cost for malaria control, which is so heavy on government would go down, would go down for householders as well*” (U14)

#### Theme 3: Governance of gene drive mosquitoes

There was widespread and persistent concern that gene drive mosquitoes are a particularly difficult technology to govern (U1-6, U8-11, U13-16, U18). Central to this concern was the *novelty and irreversibility* of gene drive mosquitoes (U1-7, U10, U13-16). The majority of participants were concerned about the novelty and potential for irreversible damage from the technology’s development and potential use that raised significant ethical concerns for governance:

“*That is a huge ethical consideration because this is something that is happening for the first time, we have never done this, we haven’t done the risk assessments that you want to release the genetically modified mosquito with gene drive into the community, what impact does it have? These are ethical issues*.” (U2)

Participants suggested that irreversibility makes risk governance particularly challenging:

“*It means we will need to clearly undertake laboratory understanding and modelling to be critically sure before even being released. Because once it comes out to the community there is no way you can take that back*.” (U15)

For some participants, novelty and irreversibility justified a careful and cautious approach to governance:

“*Sometimes it’s good to be a guinea pig. But so if we don’t have a case study [of gene drive deployment to learn from], then we have a problem that we just have to go in blind, normally you have to be cautious… It’s not like it’s a matter of life and death right now. Even if we didn’t put the gene drives here, we still have our usual way that has kept malaria down. At some point, you’re measuring, you’re weighing between our usual way which we use to control malaria and a totally new approach. You don’t rush to these new approaches*.” (U6)

Other participants cautioned against seeing gene drive mosquitoes as a technological ‘silver bullet’ to the problem of malaria. Doing so could lead to effective, less risky, alternatives being overlooked:

“*So, for me, I feel that this is an experimental technology and we should not be distracted, because there are other countries that have eradicated malaria without the need of techno fixes. I’ll give you two examples, Sierra Leone and Paraguay have eradicated malaria without gene drives. So I think we need to learn from them what they are doing right and how those lessons can be extrapolated to Uganda, yeah*.” (U10)

The second governance sub-theme to emerge is the importance of *inter*-*state relations*, concerning the international nature of gene drive development and, particularly, an inability to contain or recall gene drive mosquitoes across national borders (U1-4, U6, U9, U10, U12-14, U16-18). Two participants believed the visible success of gene drive would defuse any potential conflicts while another raised a concern that other countries may benefit from a Ugandan technology (a gene drive mosquito) without paying. However, the majority of participants were concerned that gene drive mosquitoes’ characteristics had the potential to create inter-state conflict:

“*I think the risks are not technical risks of the biological phenomenon; they are risks of cross*-*border interaction with the people… For example, you may find that a new disease has broken out in a neighbouring country which did not deploy the gene drive, and the people, through rumors, will attribute that phenomenon to the gene drive that was released*.” (U9)

For these reasons, some participants argued that a regional approach to governance was needed, supported by public engagement and acceptance:

“*Humans will need visas and passports to cross borders but mosquitoes don’t need passports, they don’t need visas to cross borders. So… if there is a field release and we will not be able to control the dispersion of these mosquitoes, modified mosquitoes, which have a gene drive attached to it, we will not be able to recall or control the trans*-*border spread … Yes, that is a genuine concern and I think it is a question of engagement, how well we engage, and then also a question of acceptance of this technology, and that cannot be predetermined*.” (U2)

The final sub-theme is *institutional uncertainty* (U1-7, U10, U13-14, U16) in which participants questioned the legal, organizational and procedural capacity necessary for gene drive governance as well as potential conflicts of interest. Some participants questioned whether Uganda had the capacity to independently appraise gene drive mosquitoes:

“*Unfortunately … we’re still trying to understand first generation GMO and gene drives is already second generation with very sophisticated technologies, like CRISPR. Our law, as it stands, I don’t think we’ll be able to regulate gene drives, as they are, because we’ve not yet even understood, we don’t even have an elaborate functioning biosafety framework.*” (U10)

Other participants pointed to the lack of scientific capacity in Ugandan institutions to adequately address novelty and complexity of gene drive:

“*The other point is on capacity, capacity within the country to actually monitor, to effectively guide the policymakers about this. Because, at any given moment the policymakers may not be scientists, or even scientists, not every scientist is a geneticist*.” (U14)

Governance concerns were compounded by a perceived lack of transparency and independence of regulators from gene drive developers:

“*The people who are promoting these technologies are the same people who want to regulate them. So I don’t think that it’s an impartial situation*.” (U10)

“*It really is still a very new concept for most of us in Uganda … Scientists may be able to share when they’re doing this but sometimes the information they share is not complete information in the sense of there are people going to be affected by this technology may not necessarily be the ones that will be consulted and they may not get an opportunity to understand how this gene drive is going to affect them … so there is a bit of scientists not being very transparent’* (U16)

Many participants called for the early involvement of a broader range of actors in governance decisions and new institutions that could address the ethical as well as the scientific risks raised by gene drive mosquitoes:

“*People really need to know what the ethics are of course what the positives, the pros and the cons that they are fully aware of, you know what is being introduced into the country*.” (U16)

“*We need a multidisciplinary committee or team that is going to regulate these technologies in the country and not just with it being seen as a scientific issue*.” (U10)

Several participants believed liability and responsibility were essential for good governance yet there was uncertainty as to whether adequate frameworks were in place:

“*First there have been issues on liability, issues to do with liability, who is liable if anything goes wrong, who takes responsibility. That has been a major concern*.” (U2)

## Discussion

This is the first study of stakeholders’ hopes for and concerns about gene drive mosquitoes as a novel approach to malaria control in Uganda, where Target Malaria is preparing for field trials. Findings reveal hopes overwhelmingly focused on reducing or eradicating malaria along with some mention of the socio-economic benefits of reducing malaria, while concerns were spread across three areas—concerns that malaria may not be reduced, possible negative impacts from the technology and questions about how the technology would be governed.

### Expanding the scope of risk regulation

High-level, collaborative governance documents prescribing risk governance frameworks for gene drive organisms recognize that engaging with social-cultural contexts is needed for the development and testing of the technology [20]. For example, Roberts et al. [12] note that definitions of biodiversity risk are dependent on ‘*what aspects of biodiversity are considered valuable*’ and call for the inclusion of values that reflect the social and cultural context. This study demonstrates that local stakeholders are able and willing to contribute relevant and important knowledge to the development of risk frameworks. For example, the ecological and human health concerns identified by the participants closely align with the risk categories identified by world-leading experts in the European Food Safety Authority (EFSA) Panel on Genetically Modified Organisms during an investigation into the adequacy of existing guidelines for the risk assessment of gene drive insects [7]. While stakeholders’ concerns aligned with EFSA risk categories, they were articulated in more detail, reflecting the local social, cultural and environmental context and helping to identify environmental protection goals.

Previous research demonstrates that stakeholders see the risks of biotechnologies more broadly than do science-based regulatory agencies, which focus almost exclusively on environmental and human health risks [32, 33]. This study shows that in addition to situating risk in the local context, stakeholders raised a broader set of concerns about gene drive mosquitoes than those considered in traditional regulatory frameworks. In particular, they are concerned about the socio-economic impacts and governance frameworks for gene drive mosquitoes. The incorporation of socio-economic considerations in regulatory frameworks is contentious and there are few examples of frameworks that have specified what counts as a socio-economic consideration or successfully integrated them into risk decision-making [32]. However, attentiveness to these considerations will be important for the ethical justifiability and social acceptability of risk decisions concerning the testing and deployment of gene drive mosquitoes [34].

### Identifying and assessing the benefits of gene drive solutions to malaria control

There is strong support from developers, funders and others for considering the potential benefits of gene drive in risk governance decisions in Africa [35]. For example, AUDA [2] promotes the importance of considering benefits and advocates the use of risk/benefit analysis in decisions about the use of gene drives which involve weighing up the human health benefits of malaria reduction or eradication against the environmental and biodiversity risks. This study shows the range of benefits that Ugandan stakeholders envisage but it also highlights a range of concerns about how these benefits might not be realized, showing their contingency. In the case of gene drive mosquitoes for malaria control in Uganda, decision-makers will have to make a trade-off between the human benefits of malaria eradication and environmental risk.

Yet while considerable empirical and theoretical work is going into assessing the risks, very little if any work is being done the assess the benefits in a substantive way. For example, Carter and Friedman [36] state: ‘*The benefits are clear if these research efforts are successful; however, the risks must first be carefully evaluated*’ and a more recent report argues ‘*In spite of all of these valuable benefits, there must be careful consideration of risks associated with gene drives*’ [37]. Each of these examples takes the benefits as self-evident. However, as with risks, benefits (and the conditions that are necessary to realize them) will need to be considered in the local context. That is, effort needs to be made to identify the benefits that could, or are likely to result, and also assess the social, political, economic, ecological and technical conditions that need to occur for those benefits to be realized. Without such consideration, technical optimism may make the assumed benefits seem more likely than they are.

Underlying the call by developers and funders to consider potential benefits is the perception that a focus on assessing risks without considering benefits will favor restrictive field testing designs [38]. There is a danger that given the huge investments in regulation development by the BMGF and other philanthropists, benefits will be included in order to reduce the risk of restrictive regulations and facilitate the deployment of the technology. For example, in 2020, AUDA received US$6 million to enable the deployment of gene drive mosquitoes for malaria control and in 2016, the US Foundation for the National Institutes of Health received US$8.3 million to ‘accelerate’ preparations for gene drive mosquitoes in Africa [27]. These top-down pressures to facilitate and support gene drive make the degree to which Ugandan voices can be heard more challenging and may make stakeholder hopes for technology and risks of not realizing these benefits less visible.

However, international and regional developments can be complemented by the involvement of national stakeholders who live with malaria on a daily basis and are knowledgeable actors who possess relevant expertise needed to contribute to benefit assessment and the exploration of pathways to realize such benefits.

The assessment of benefits and risks is inherently a value-based exercise and there are powerful arguments for opening up risk decision-making to local actors on these grounds in addition to the substantive reasons discussed above [35, 39]. Decisions which balance benefits to human health against possible environmental harms, particularly in comparison to other possible solutions with fewer risks (i.e. bed nets) are ethical decisions and cannot be made without ethical analysis and engagement with local communities [40]. Overall, a more integrated approach, one that integrates the risks and benefits as well as the science and the politics, may help to clarify the decisions to be made and the trade-offs between the risks and benefits of gene drive solutions to malaria.

### New directions for inclusive risk governance processes

An important finding from the study is that the way in which gene drive mosquitoes are governed is a source of concern for stakeholders. There was a great deal of uncertainty about whether existing institutions and governance frameworks were sufficient and what the alternatives might be. A decision-making pathway is needed for gene drive mosquitoes, clarifying in particular the role risk assessment will play and how potential benefits and socio-economic factors will be considered in decision-making. If decisions are based on narrow risk assessments conducted by experts, particularly if these have been developed by non-Ugandans, then there is a high risk that the decision will be perceived by Ugandan stakeholders and/or members of the public as illegitimate. A decision-making pathway is needed that draws on local knowledge and values, garnered through engagement with stakeholders, publics and communities and it should not rely on arm’s length public consultations on highly technical risk guideline documents produced solely through expert-centered processes. International support is available through the Cartagena Protocol on Biodiversity which allows States to consider societal concerns such as the ethical, social, and economic impacts of gene drive mosquitoes in regulatory decisions [32].

Not surprisingly, there is a demonstrable willingness on the part of communities and publics to engage with projects which may reduce or eliminate malaria [3, 4]. Further, involving local actors helps to build much-needed regulatory and risk decision-making capacity [6, 10]. However, engaging with national stakeholders may be seen as a distraction from the expert-led risk governance activities currently underway at the regional and international level. Importantly, local actors are significantly less resourced, making it more difficult for their voices to be heard, particularly when they may compete with regional African Union-led efforts to harmonize risk regulatory frameworks which are well-funded by gene drive funders and embody powerful beliefs about the importance of technology for Africa’s development (Hartley et al, pers. commun.). However, stakeholder and community input into risk governance processes will help to ensure that the development and deployment of the technology is conducted in a democratic and deliberative fashion. Despite the recognition that traditional forms of engagement in risk regulatory processes will not be sufficient for gene drive mosquitoes and calls for rigorous stakeholder engagement in regulation and oversight [35, 38], there is a lack of concrete thinking in current expert-led risk governance activities about how and when stakeholders, communities and/or publics can provide input and shape the regulatory frameworks that will be designed to protect them. Recent experiences with regulatory decisions to release genetically modified insects in the USA, Brazil and Burkina Faso fail to demonstrate meaningful or rigorous stakeholder engagement in both the development of risk governance frameworks and case-by-case decisions on individual releases [41–43].

## Conclusion

Malaria is a global health priority [44]. Aside from the obvious and severe health implications, malaria affects population growth, saving and investment, worker productivity, absenteeism from school, trade and foreign direct investment, premature mortality, and medical costs [44]. Gene drive mosquitos are a cutting-edge and promissory approach to vector control for malaria control and eradication, which are currently supported by international and regional actors such as the BMGF, AU and the AU’s High-Level Panel on Emerging Technologies. To prepare for gene drive mosquito field trials, an international process is underway to develop a risk governance framework. This framework is important as it will outline the way in which decisions about whether or not to release gene drive mosquitoes will be made and is expected to shape national and regional risk decision-making.

Social science research tells us that externally imposed, top-down and locally-insensitive approaches to technology introduction and development in Africa are problematic and can limit a technology’s potential to serve its intended goals [45, 46]. International processes to develop risk governance frameworks can provide high-level guidelines but risk decision-making must be grounded in the local context and involve local stakeholders, communities and publics if it is to be robust, meaningful and legitimate. This type of engagement should accompany and complement (not replace) the community engagement work necessary for gaining a community’s prior, free and informed consent to participate in or host field trials [47]. In a modest step, this study brings Ugandan voices into the discussion about the hopes and concerns for gene drive mosquitoes which are planned to be released in field trials in Uganda. The data shows the importance of assessing the benefits and socio-economic impacts in collaboration with stakeholders and communities, and delineating a clear decision-making pathway that includes engagement, conducted by regulators and decision-makers and recognizing the value decisions involved. Decisions about whether or not to release gene drive mosquitoes will need to consider the assessment of both the risks and the benefits of gene drive mosquitoes for malaria control within a particular social, political, ecological, and technological context.

## Data Availability

The datasets generated during and/or analyzed during the current study are available in the UK DATA SERVICE repository, https://ukdataservice.ac.uk/. The datasets during and/or analyzed during the current study are also available from the corresponding author on reasonable request.
